# Pesticide exposure and adverse health effects associated with farmwork in Northern Thailand

**DOI:** 10.1002/1348-9585.12222

**Published:** 2021-05-11

**Authors:** Chanese A. Forté, Justin Colacino, Katelyn Polemi, Andrea Guytingco, Nicholas J. Peraino, Siripond Jindaphong, Tharinya Kaviya, Judy Westrick, Richard Neitzel, Kowit Nambunmee

**Affiliations:** ^1^ School of Public Health Environmental Health Sciences University of Michigan Ann Arbor MI USA; ^2^ College of Engineering Scientific Computing University of Michigan Ann Arbor MI USA; ^3^ School of Public Health, Nutritional Sciences University of Michigan Ann Arbor MI USA; ^4^ School of Medicine Center for Computational Medicine and Bioinformatics University of Michigan Ann Arbor MI USA; ^5^ Department of Chemistry Wayne State University Detroit MI USA; ^6^ School of Health Science Mae Fah Luang University Chiang Rai Thailand

**Keywords:** epidemiology, industrial medicine, occupational medicine, public health, toxicology

## Abstract

**Objectives:**

To assess pesticide exposure and understand the resultant health effects of agricultural workers in Northern Thailand.

**Methods:**

This was a cross‐sectional study. We quantified exposure to pesticides, including chlorpyrifos, methomyl, and metalaxyl, by air sampling and liquid chromatography/mass spectrometry. We estimated differences in self‐reported health outcomes, complete blood counts, cholinesterase activity, and serum/urine calcium and creatinine concentrations at baseline between farmworkers and comparison workers, and after pesticide spraying in farmworkers only.

**Results:**

This study included 97 men between the ages of 22 and 76 years; 70 were conventional farmworkers; and 27 did not report any prior farmwork or pesticide spraying. None of the farmworkers wore standardized personal protective equipment (PPE) for the concentrated chemicals they were working with. Methomyl (8.4‐13 481.9 ng/m^3^), ethyl chlorpyrifos (11.6‐67 759 ng/m^3^), and metalaxyl (13.9‐41 191.3 ng/m^3^) were detected via personal air sampling. When it came to reporting confidence in the ability to handle personal problems, only 43% of farmworkers reported feeling confident, which reflects higher stress levels in comparison to 78% of comparison workers (*P* = .028). Farmworkers also had significantly lower monocyte counts (*P* = .01), serum calcium (*P* = .01), red blood count (*P* = .01), white blood cell count (*P* = .04), and butyrylcholinesterase activity (*P* < .0001), relative to comparison workers. After adjusting for body mass index (BMI), age, and smoking, methomyl air concentrations were associated with a decrease in farmworker acetylcholinesterase activity (beta = −0.327, *P* = .016).

**Conclusions:**

This population of farmworkers had significant alterations in stress measures and clinical biomarkers, including decreased blood cell counts and cholinesterase activity, relative to matched controls. These changes are potentially linked to occupational pesticide exposures. Improving PPE use presents a likely route for preventive intervention in this population.

## 1 INTRODUCTION

Agriculture is a major contributor to Thailand's economy.^1^, ^2^ Among surveyed farmers residing in Northern Thailand, most (97%) reported using pesticides on their crops.^3^, ^4^, ^5^, ^6^ Over 93% of agriculture workers in Thailand work through the informal sector and are not defined as employees under the Labor Protection Act; thus, these workers are exempt from numerous safety laws surrounding labor and social security rights.^7^, ^8^ Thailand ranked third out of 15 Asian countries for pesticide use per unit area and fourth in annual pesticide use.^2^ Although there have been increases in organic food consumption in Thai markets, there is no evident reduction in pesticide use.^2^, ^7^ In fact, pesticide use in Thailand has increased from 110 000 tons in 2007 to roughly 172 000 tons in 2013.^2^ For the pesticides being imported into Thailand, a third are considered extremely, highly, or moderately hazardous based on the World Health Organization's (WHO) hazard categories.^2^, ^9^

Pesticide pollution in the environment is associated with poisonings, oxidative stress, neurological dysfunction, birth defects, reproductive disorders, metabolism disorders such as diabetes mellitus, and cancers including colorectal cancer, prostate, and non‐Hodgkin lymphoma.^8^, ^10^, ^11^, ^12^, ^13^, ^14^ Although Thailand does not have a poisonings registry, a poison center located in Bangkok recorded more than 15 000 patients over a 3‐year period, 42% of whom had poisonings related to pesticide exposure.^8^ The pesticides most associated with these poisonings were insecticides: carbamates, organophosphates, and pyrethroids.^8^ Research is lacking on low‐ and middle‐income countries (LMIC) pesticide exposure and related health outcomes.^15^

This study was motivated by a group of contract farmers residing in Wiang Pa Pao, Chiang Rai, Thailand. These farmers reported concerns about their health related to spraying pesticides to Chiang Rai Prachanukroh Hospital employees. Our project was launched in response to this concern. The purpose of this project was to assess the pesticide exposure of these farmworkers in Northern Thailand and to understand the resultant health effects when compared with workers residing in a non‐agricultural area (Chiang Rai, Thailand). This project adopted a mixed‐methods approach during assessment of pesticide exposure and the resulting health effects including (a) personal air sampling, (b) biomarker sampling, and (c) perceived exposure and health effects assessed by questionnaire.

## 2 MATERIALS AND METHODS

The STROBE cohort reporting guidelines and checklist were completed in the creation of this report. The study protocol was approved by the Institutional Review Boards of Mae Fah Luang University and the University of Michigan (UM Submission ID: HUM00128091/CR00068767).

This project was initiated because farmworkers who were seeking care from the regional health care center repeatedly requested a study be done on their health in relation to their work. The local community, village leaders, and healthcare volunteers (laypersons with healthcare training) were imperative to the creation and completion of this research project. These community members were consulted and paid in kind for their expertise in identifying and communicating with stakeholders for the study, coordinating transportation, participant recruitment and engagement, data collection, and translation between English and Thai (two dialects of Thai included in this study).

### 2.1 Study population

All study participants were 21 years of age and older, male, and resided in Northern Thailand. Additionally, all farmworkers contracted with the same international food company at the time of the study, because the consent of the company was also requested. All farmworkers were also working as pesticide sprayers either part‐time or full‐time at the time of the study. Because this study was initiated from the farmworkers, most farmworkers and comparison workers were recruited through word of mouth. Each participant provided oral consent to participate in the study, which was noted by the researcher administering the survey in the first step of study participation. Participants were recruited in Chiang Rai, Thailand, using a recruitment script administered (in Thai) by health volunteers—laypersons trained through the Chiang Rai Prachanukroh hospital on patient care and interactions. This script included background information on the study, what to expect as a participant of the study, and more importantly fully defined consent to make it clear to the participants they have the right to stop or deny participation at any point. This recruitment script also included information on compensation and that all participants, no matter their sampling consent, will receive compensation for enrolling and taking the researcher administered survey.

Comparison workers were recruited through word of mouth at Mae Fah Luang University (MFU), with a focus on older males in work fields unrelated to agriculture with no commercial pesticide spraying experience. Farmworkers and comparison workers were recruited between July 1, 2016, and September 15, 2016. Overall, there were 97 study participants recruited and examined for eligibility. Twenty‐seven comparison workers and 20 farmworkers were retained in the study who met the following inclusion criteria: were 21 years or older, male, residing in Northern Thailand, and completed all follow up.

### 2.2 Sample collection

All participants completed the consent form by oral confirmation due to literacy differences. Conventional farmworkers consented to allow work observation, active air sampling, and pre‐ and post‐spray urine, and blood samples to be taken. Each study participant also completed a researcher‐administered survey. Participants received 600 Thai baht (approximately 20 US dollars) for participation and completion of the study.

We quantified exposure and potential adverse health effects among workers by self‐report, biological, and environmental sampling. A 69‐item occupational health questionnaire was translated into Thai from English by MFU researchers. The questionnaire was based on a previous study of worker health and occupational noise.^16^ The survey was administered to study participants at the local community center and took roughly 35‐45 minutes to complete. Questionnaire data were collected from July 2017 to the end of August 2017 (conventional farmworkers and comparison workers) and again in January 2018 (sample of comparison workers from Chiang Rai).

Nurses collected 10 mL of urine and 10 mL of blood from workers at the local hospital (Chiang Rai or Wiang Pa Pao). For farmworkers, blood and urine samples were collected within 1 week prior to pesticide spraying and again within 3 days after pesticide spraying. Whole blood samples were collected in red‐top tubes with no anticoagulant, lavender‐top tubes with EDTA, and green‐top tubes with heparin.

### 2.3 Blood and urine analysis

Urine creatinine, urinary calcium, serum creatinine, serum calcium, and complete blood counts (CBCs) were quantified by Meng Rai Laboratory in Chiang Rai, Thailand. Acetylcholinesterase (AChE) and butyrylcholinesterase (BuChE) were analyzed using the Ellman method, from whole blood and serum, respectively.^17^ AChE was analyzed because it is directly related to AChE inhibition by the pesticides, and BuChE because it can be a sensitive biomarker of exposure to AChE inhibitors.^18^ Concentrations of the health biomarkers were compared between conventional farmworkers and comparison study participants, as well as within farmworkers before and after spraying.

### 2.4 Air sample collection and analysis

Personal pesticide air exposure levels were measured in conventional farmworkers only. GilAir Plus Personal Air Sampling Pumps by Sensidyne, Inc were calibrated each day with the Gilibrator Calibrator before heading to the field. Air samples were collected during the time farmers were spraying pesticides (14‐63 minutes) using XAD‐2 sorbent tubes (SKC Ltd) based on the standard NIOSH method of 5600 at a flow rate of 1 L/min.^19^ Field blanks were collected by opening tubes away from the farms for a similar period of time; no air was drawn through the blanks, but they were otherwise handled identically to actual air samples.

The analysis of pesticide residues was completed by the Lumigen Instrument Center at Wayne State University, and researchers were blinded to exposure or worker category. Laboratory controls from a spiked and blank filter were extracted each batch. A calibration check at 10 ng/mL and a solvent blank were run every 10 samples. If a check did not pass within 20% error, the entire 10 sample section was re‐run. Calibration curves were prepared the same day per batch by comparing the concentration of and area ratio of analytes to deuterated surrogates. A linear regression was taken, and percent error was calculated at each calibration point. If a point did not fall within 20% error of the predicted value, it was dropped from the end of the curve.

Air sampling tubes were extracted as either top portions or bottom portions. Top portions of the tubes contained the retainer ring, filter paper, foam pad, and XAD fill. Bottom portions contained the middle foam pad, and the bottom portion of XAD fill; the bottom foam was discarded. Portions were transferred to a 2 dram vial followed by 1960 μL of methanol and 40 μL of a solution containing 1 μg/mL each of deuterated surrogates. The vials were tightly capped and sonicated for 30 minutes, allowing the bath to heat naturally. Heating was found necessary to achieve equilibration of the surrogates and standards to the XAD fill and improve recoveries. Samples were centrifuged to settle any particulates and 900 μL of sample was diluted with 100 μL of water containing 100‐mmol/L ammonium formate, resulting in a final sample containing 10% water and 10‐mmol/L ammonium formate. Liquid chromatography mass spectroscopy (LCMS) analysis was completed using a Nexera‐X2 ultra performance liquid chromatography (UPLC) with Shimadzu 8040 triple quadrupole mass analyzer. Chromatography was achieved using a Waters acquity UPLC Ethylene‐Bridged Hybrid (BEH) C18 (1.2 μm, 2.1 × 50 mm) column eluting with 10‐mmol/L ammonium formate in water (Mobile Phase A) and 10‐mmol/L ammonium formate in methanol (Mobile Phase B).

### 2.5 Worker observations

MFU and UM student researchers observed the farmworkers during spraying activities and recorded worker practices for mixing, spraying, and storage of the pesticides on a worker observation form. This included information on the pump used, pump calibration, personal protective equipment (PPE) used, and notes on common practices. Detailed notes on PPE used such as item and material were noted by observers (eg gloves made of rubber, latex, or cloth). Work observations were not completed for comparison workers because they do not perform commercial pesticide spraying.

### 2.6 Statistical analyses

For questionnaire data, we calculated summary statistics of demographic data across the entire population. We first tested for crude differences across the measures between the conventional farmers and the comparison workers by t test for continuous variables or by Fisher's exact test for categorical variables. Analysis of variance (ANOVA) and multivariate regression analyses were used to compare serum and urinary biomarker concentrations between farmworkers and comparison workers, adjusting for the potential confounders age, smoking status, alcohol consumption, and body mass index (BMI). We also conducted multivariate linear regression analyses of the association between pesticide levels quantified in the personal air samples and serum and urinary biomarkers in the farmworkers collected after spraying, adjusting for the same covariates as described above. Any variables that were not normally distributed were log transformed prior to regression. SAS 9.4 was used to complete regressions and demographic tables, and R 3.5.1 was used to create figures and graphics.

### 2.7 Data statement

The de‐identified data can be made available via Dropbox upon reasonable request and agreement to a memorandum of understanding of ethics.

## 3 RESULTS

Initially we recruited 73 farmworkers and 29 healthcare workers, and upon further recruitment, we were able to secure more general comparison workers through word‐of‐mouth recruitment at Mae Fah Luang University. This new general comparison worker group was comprised 27 men with similar mean age and education backgrounds as the farmworkers. Ultimately, the healthcare workers were overly female and had college educations and were dropped. The 27 comparison workers and the 70 male farmworkers were retained for this study.

Overall, farmworkers and comparison workers had similar demographics, except for BMI, which was significantly lower for farmworkers (Table 1). Farmworkers had a median age of 50 years with a range of 22‐76 years of age, and comparison workers had a median age of 49 with a range of 39‐68 years. Most of the workers were married, attended primary and secondary school, drank alcohol 1‐3 d/wk, and were current smokers (Table 1).

**TABLE 1:** Study participant demographics, males only

Variable	Variable outcome	Comparison worker		Farmworker		Fisher's exact significance
		N = 27	%	N = 70	%	*P* value
Marital status	Single	6	22.2	7	10.0	.11
	Married	16	59.3	55	78.6	—
	Divorced	3	11.1	2	2.9	—
	Living with partner	2	7.4	6	8.6	—
Education	None	0	—	3	4.3	.48
	Primary	18	66.7	49	70.0	—
	Secondary	6	22.2	15	21.4	—
	Some college	1	3.7	2	2.9	—
	4 year degree	1	3.7	1	1.4	—
	Graduate level	1	3.7	0	—	—
Alcohol	Never	7	26.9	12	17.4	.64
	Not much	4	15.4	14	20.3	—
	1‐3 d/wk	9	34.6	18	26.1	—
	4‐6 d/wk	4	15.4	14	20.3	—
	Daily	2	7.7	11	15.9	—
Tobacco use	Never smoked	8	30.8	25	35.7	.67
	Former smoker	8	30.8	15	21.4	—
	Currently smokes	10	38.5	30	42.9	—
		**Median**	**SD**	**Min.**	**Max.**	
Comparison worker age		49.0	8.1	39.0	68.0	.61
Farmworker age		50.0	11.0	22.0	76.0	—
Comparison worker BMI		24.7	3.7	20.0	34.1	.02^a^
Farmworker BMI		22.2	3.5	16.1	33.1	—

Abbreviation: BMI, body mass index.

^a^ Signifies a *P* value less than .05.

Based on worker observations, none of the farmworkers wore chemical proof aprons, chemical proof gloves, or a respirator. The farmworkers did wear an item that we named “face hats,” which included both home‐made and commercial versions. The home‐made version was usually two towels or scarves (eg, durags) stitched together to cover the head and face, and the commercial version was a canvas brimmed hat with a canvas face covering that could be removed. The use of gloves, long sleeve clothing, and any sort of clothes covering (eg, rain poncho or plastic sheet) were usually used items with some damage, and use was not consistent across workers.

With respect to self‐reported health concerns, farmworkers and comparison workers self‐reported symptom responses only statistically significantly differed for “dizziness” and “shaking or trembling of hands” (Table S2). Table S3 presents worker self‐reported stress levels by worker category based on the Cohen's Perceived Stress Scale. Overall, comparison workers more frequently reported stress in comparison with farmworkers, although not statistically significantly so. However, when it came to reporting confidence in ability to handle personal problems, only 43% of farmworkers reported feeling confident often in comparison with 78% of comparison workers (*P* value = .03).

**FIGURE 1:**
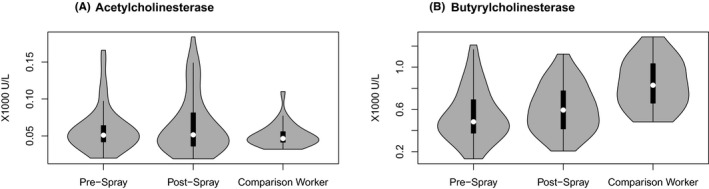
A and B, Distribution of acetyl‐ and butyrylcholinesterase activities in comparison workers (one time point only) and farmworkers (pre‐ and post‐spray). In previous research completed in Northern Thailand, the AChE national reference range is 6400‐8200 U/L^36^ Sapbamrer et al^32^ N = 97

A comparison of cholinesterase activity between the two groups indicated that although there were some farmworkers with higher AChE activity, the AChE levels in farmworkers and comparison workers were not significantly different (*P* = .20; Figure 1A). Comparison workers had higher BuChE activity levels compared with farmworkers (*P* < .0001; Figure 1B). AChE and BuChE concentrations were not correlated (Pearson = −0.09, *P* = .35, 95% confidence interval [CI] [−0.29, 0.10]). Within farmworkers only, pre‐spray and post‐spray activity of AChE (0.32, *P* = .01) and BuChE (0.31, *P* = .01) were correlated.

**FIGURE 2:**
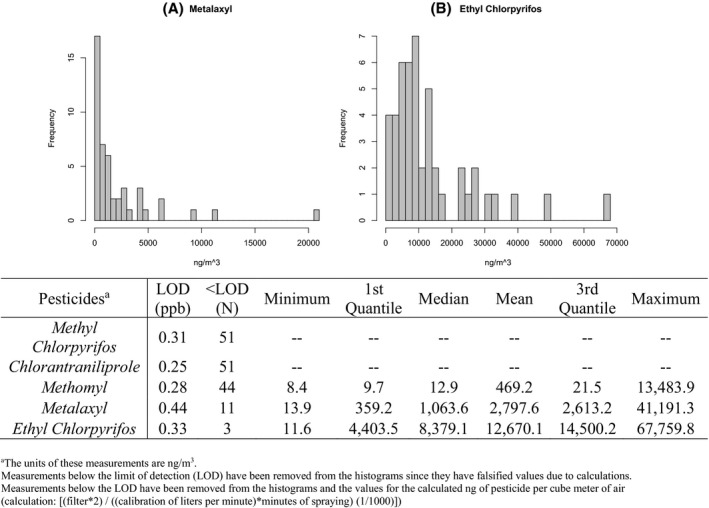
Pesticide measurements captured by personal air monitors on Northern Thailand farmworkers (N = 51) with histograms showing distributions of detected values of (A) metalaxyl and (B) ethyl chlorpyrifos. ^a^The units of these measurements are ng/m^3^. Measurements below the limit of detection (LOD) have been removed from the histograms because they have falsified values due to calculations. Measurements below the LOD have been removed from the histograms and the values for the calculated ng of pesticide per cube meter of air (calculation: [(filter*2) / ((calibration of liters per minute) * minutes of spraying) (1/1000)])

Measurements of pesticide residues on air samples are displayed in Figure 2. Overall, ethyl chlorpyrifos and metalaxyl were detected at the highest frequency, while methomyl was less frequently detected. Most chlorantraniliprole (N = 60) and methyl‐chlorpyrifos (N = 61) measurements were below the limit of detection. Ethyl chlorpyrifos, followed by metalaxyl and methomyl, was found in the highest concentrations in the air filter samples.

Table 2 presents linear regression parameter estimates and CIs comparing biomarker concentrations between farmworkers and comparison workers. Eosinophil (*P* = .02), urine creatinine (*P* = .03), and mean cell volume (MCV) (*P* = .04) concentrations were statistically significantly higher in farmworkers than the comparison group. Monocytes (*P* = .01), red blood cell counts (*P* = .01), white blood cell count (*P* = .04), and serum calcium (*P* = .02) were statistically significantly lower in farmworkers than comparison workers.

**TABLE 2:** Linear regression analyses comparing biomarker concentrations in farmworkers and comparison workers

Analytes	Units	Farmworkers = 1 vs. comparison = 0 N = 97							
		Unadjusted values				Adjusted values			
		β estimate	*P* value	95% CI		β estimate^b^	*P* value	95% CI	
log(Basophil)	%	0.08	.25	0.23	−0.06	0.12	.13	−0.04	0.29
log(Eosinophil)	%	0.19	.05	0.00	0.37	0.24^a^	.02	0.04	0.44
log(Hemoglobin)	g/dL	−0.02	.06	−0.03	0.00	−0.01	.16	−0.03	0.01
log(Hematocrit)	%	−0.01	.15	−0.03	0.00	−0.01	.28	−0.03	0.01
log(Lymphocyte)	%	−0.04	.25	−0.10	0.03	−0.03	.41	−0.10	0.04
log(Mean corpuscular hemoglobin concentration [MCHC])	pg	0.00	.37	−0.01	0.00	<0.01	.53	−0.01	0.01
log(Mean corpuscular volume)	Fl	0.03	.02	0.00	0.06	0.03^a^	.04	<0.01	0.06
Monocyte	%	−1.09	.02	−1.99	−0.20	−1.33^a^	.01	−2.30	−0.37
Neutrophil	%	−0.48	.87	−6.49	5.53	−1.34	.69	−8.08	5.39
log(Plate count)	cells/μL	−0.02	.52	−0.09	0.04	−0.03	.39	−0.10	0.04
log(Red blood cell [RBC] count)	cells/μL	−0.04	.00	−0.08	−0.01	−0.04^a^	.01	−0.07	−0.01
log(RBC distribution width [RDW])	%	0.01	.15	−0.01	0.03	0.01	.19	−0.01	0.03
log(White blood cell count)	cells/μL	−0.07	.01	−0.12	−0.02	−0.06^a^	.04	−0.12	<0.01
Serum calcium	mg/dL	−0.83	.00	−1.31	−0.35	−0.79^a^	<.01	−1.31	−0.27
log(Serum creatinine)	mg/dL	−0.04	.04	−0.08	0.00	−0.03	.22	−0.07	0.02
log(Urine calcium)	mg/dL	0.13	.12	−0.03	0.30	0.14	.14	−0.05	0.32
Urine creatinine	mg/dL	33.03	.03	3.82	62.24	36.29^a^	.03	4.17	68.41
log(Acetylcholinesterase)	U/L	0.08	.37	−0.10	0.27	0.05	.64	−0.16	0.25
log(Butyrylcholinesterase)	U/L	−0.48	<.01	−0.68	−0.29	−0.50^a^	<.01	−0.70	−0.29

Abbreviation: CI, confidence interval.

^a^ Signifies a *P* value less than .05.

^b^ These values were adjusted for age, former smoker status, current smoking status, alcohol use, and body mass index.

Table 3 presents the adjusted linear regression beta estimates between biomarker concentrations and air sample pesticide content for farmworkers only, and Table S1 presents these same data unadjusted for confounders. The ratio of pre‐ and post‐spray AChE was significantly lower for increased concentrations of methomyl in air samples (*P* = .02). Urinary creatinine and serum calcium were inversely associated with air sample concentrations of metalaxyl.

**TABLE 3:** Linear regression models of association between blood analytes and pesticide air sample concentrations (N = 51)

Analytes	Units	Log(Methomyl)^c^				Log(Metalaxyl)^c^				Log(Ethyl chlorpyrifos)^c^			
		β	*p* value	95% CI		β	*p* value	95% CI		β	*p* value	95% CI	
log(Basophil)	%	0.28	.52	−0.61	1.16	−0.19	.05	−0.39	0.00	−0.03	.83	−0.30	0.25
log(Eosinophil)	%	−0.25	.64	−1.33	0.84	−0.07	.56	−0.33	0.19	−0.22	.17	−0.54	0.10
log(Hemoglobin)	g/Dl	−0.01	.79	−0.09	0.07	−0.01	.41	−0.03	0.01	−0.01	.40	−0.03	0.01
log(Hematocrit)	%	−0.02	.65	−0.11	0.07	0.00	.80	−0.02	0.01	−0.01	.38	−0.04	0.02
log(Lymphocyte)	%	0.18	.33	−0.19	0.54	0.00	.94	−0.09	0.09	−0.08	.15	−0.19	0.03
log(MCHC)	pg	0.01	.68	−0.04	0.06	−0.01	.37	−0.02	0.01	0.00	.83	−0.01	0.02
log(MCV)	Fl	−0.07	.28	−0.22	0.07	0.02	.27	−0.02	0.05	0.01	.50	−0.03	0.06
Monocyte	%	−1.38	.58	−6.49	3.73	0.32	.59	−0.91	1.55	0.18	.82	−1.40	1.76
Neutrophil	%	−1.61	.93	−38.75	35.53	0.93	.83	−7.98	9.83	6.35	.24	−4.69	17.40
log(Plate count)	cells/μL	0.13	.37	−0.16	0.41	0.00	.98	−0.07	0.07	−0.07	.12	−0.15	0.02
log(RBC)	cells/μL	0.05	.49	−0.11	0.22	−0.02	.26	−0.06	0.02	−0.03	.27	−0.07	0.02
log(RDW)	%	0.05	.26	−0.04	0.14	0.00	.86	−0.02	0.03	0.00	.93	−0.03	0.03
log(WBC)	cells/μL	−0.12	.31	−0.37	0.12	0.01	.83	−0.05	0.07	−0.02	.62	−0.10	0.06
Serum calcium	mg/dL	0.13	.62	−0.38	0.63	−0.55^a^	.02	−1.02	−0.08	0.18	.59	−0.48	0.83
log(Serum creatinine)	mg/dL	−0.02	.20	−0.06	0.01	−0.01	.55	−0.05	0.03	0.03	.22	−0.02	0.08
log(Urine calcium)	mg/dL	0.02	.81	−0.13	0.17	−0.05	.52	−0.19	0.10	0.07	.45	−0.12	0.26
Urine creatinine	mg/dL	6.39	.64	−20.60	33.38	−30.17^a^	.02	−54.94	−5.41	5.70	.74	−29.21	40.61
AChE ratio^b^	U/L	−0.37^a^	.02	−0.66	−0.07	0.05	.72	−0.25	0.36	0.35	.08	−0.04	0.74
BuChE ratio^b^	U/L	−0.13	.35	−0.40	0.15	−0.05	.70	−0.33	0.22	−0.12	.50	−0.48	0.24

Abbreviation: CI, confidence interval.

^a^ Signifies a *P* value less than .05.

^b^ Cholinesterase ratios were calculated by dividing post measures by pre measures.

^c^ These values were adjusted for age, former smoker status, current smoking status, alcohol use, and body mass index.

## 4 DISCUSSION

Pesticide toxicity can be either acute, sub‐chronic, or chronic toxicity; with acute being a single large‐dose exposure incident, sub‐chronic being multiple exposures over weeks or a few months, and chronic toxicity being multiple exposures over several months or years.^20^ Different types of toxicity can result in different symptoms, and usually, the timing of the symptoms are much closer for acute toxicity, and for chronic exposures, toxicity is usually delayed from exposure.^20^ Farmworkers are exposed to pesticides via three pathways: dermal contact, ingestion, and inhalation.^20^

Most pesticide research studies include information on farmworker pesticide application at three stages: mixing and loading the pesticides, applying the pesticide spray solution, and cleaning up of the spraying equipment similar to the study presented in this article.^20^ The study presented in this article observed farmworkers at these three stages of pesticide application in accordance with this. However, there are techniques for reducing exposure pathways such as wearing PPE to protect the skin, mouth, and eyes; not using power equipment, which can reduce aerosolized pesticide; spraying outside to reduce confined space with the pesticide exposure; and storing pesticides in their original packaging.^20^ Pesticides exposure can cause blindness, vomiting, or even death.^20^

### 4.1 Perceptions of risk and PPE use

Our pilot study did not capture data on perceived risk of pesticide spraying and PPE use, but this is something important to consider when looking at use. In a cohort study of cotton farmers in Greece, older growers (mean age of 59.0 ± 3.0 years) were statistically significantly more likely to agree with that harmful pests on their crops were a larger concern than the chemicals used to get rid of them (*t* = 4.48, *P* ≤ .01) when compared with younger growers (mean age 27.8 ± 4.9 years).^21^ Additionally, the growers in this study almost never had access to a respirator either, and although still rarely having access to a face mask, goggles, or coveralls, younger farmers significantly had more access than older growers (*t* = 8.17, *t* = 7.05, *t* = 5.06, *P* ≤ .01).^21^ In a study of pesticide operators in Greece, most of the farmworkers showed unsafe behavior (66.1%), and the majority perceived pesticides as low risk (65.2).^21^

In a study of Iranian farmworker perceptions and PPE use, farmworkers perceived the importance of PPE much higher than the use of PPE and more specifically most of these farmworkers also had little to no respirator use.^22^ Many workers ranked the importance of not eating, drinking, or smoking during pesticide application, but some workers still admit to doing so while applying pesticides.^22^ In a study on cotton farmers in Northern Greece, PPE use varied depending on the item of clothing with boots and hats being the most common PPE used.^23^ While the overwhelming majority of farmworkers reported almost never wearing a respirator, less than 5% reported almost always or occasionally wearing a respirator suggesting that the farmworkers may have access to respirators.^23^ In a cohort of Iranian apple farmers, 8% of the 200 farmers reported also preparing pesticides in the kitchen.^24^ Overall, apple farmers reported often following safety behaviors like washing hands with soap after spraying (mean = 4.8 out of 5, SD = 0.3), not eating or drinking while spraying (mean = 4.8 of out 5, SD = 0.4), and not smoking during spraying.^24^ Cuenca et al studied 257 farmers (44% women and 56% men) in three different communities in Bolivia for PPE use and health outcome concerns of workers.^25^

Overall, farmers in more tropical regions used organophosphates more than the other communities.^25^ A minority (40%) of Bolivian farmers had at least one article of clothing that they could use as PPE, and only 17% of the farmers were well protected with PPE.^25^ The majority of farmers (80%) reported feeling sick after spraying pesticides.^25^ Additionally, Cuenca et al found that headache (80% of women and 70% of men) and dizziness (29% of women and 46% of men) were the most often reported symptoms related to pesticide spraying activities among the farmers.^25^ While we did not measure perceptions of risk and PPE use among our farmworkers, because the study was initiated by the farmworkers, it is possible they may perceive pesticide as a higher risk or more harmful than these workers. To better understand perceptions and the needs of the farmworkers, a focus group among these farmworkers should be performed.

Greek farmworkers' perceptions also show the importance of personal safety, and safe behavior were not a priority for most workers (with only 44.5% and 41.1%, respectively).^26^ Additionally, in another study in Pakistan on women farmworkers' health, researchers found that things like illiteracy, frequency of illness, medical treatment, and traditional treatment were negatively associated with PPE use.^27^ In this study, the farmworkers initiated contact with the research team; therefore, we hypothesize that these Thai farmworkers may be more concerned with safety and behavior than the farmworkers from these studies. Understanding farmworker perceptions of risk, and how these vary across different cultural and demographic groups, will be essential for designing interventions to encourage PPE use and safe handling of pesticides.

### 4.2 Cholinesterase

Other studies have assessed the relationship between pesticide exposure and cholinesterase activity outside of Thailand. Strelitz et al measured baseline to post‐spraying whole blood and serum cholinesterase levels of 215 farmworkers from the Washington State Cholinesterase Monitoring Program.^18^ Ellman colorimetric enzymatic assays were used by two different laboratories to measure BuChE and AChE using the same methodology (Washington State Public Health Laboratories in 2006 and Pathology Associates Medical Laboratories in 2007‐2011).^18^ In this same study, cholinesterase depression was defined as 20% or greater decrease from baseline to post‐exposure cholinesterase exposure.^18^ The authors found AChE and BuChE activity to be negatively correlated (−0.14, 95% CI [−0.27, −0.01]).^18^ Our study also found the correlation between AChE and BuChE to be weak, but the correlation was non‐significantly positive (0.05, 95% CI [−0.20, 0.29]). The ratio of pre‐ and post‐ spray AChE activity was also significant lower with increasing concentrations of methomyl in air samples. Others have reported BuChE activity as a more sensitive biomarker of exposure to cholinesterase inhibitors than AChE.^18^, ^28^, ^29^ In our study, BuChE activity was significantly lower in conventional farmworkers relative to comparison workers.

A cross‐sectional study in greenhouse and packinghouse workers residing in Ethiopia also used the Ellman method to analyze serum cholinesterase. In total, this study looked at 588 flower farmworkers.^30^ This study included 311 women who work in the greenhouse (n = 156) or the packinghouse (n = 155).^30^ When completing a t test between sprayers and non‐sprayers, there was not a significant difference in BuChE outcomes (25.9 vs. 24.2, *P* = .85).^30^ Most of their farmworkers used a half face respirator mask, gloves, boots, and overalls (with one farm having textile only and not chemical proof overalls.^30^ The chemicals sprayed by the sprayers were predominantly un‐registered chemicals (n = 45, 29.2%).^30^ These farmworkers sprayed numerous types of pesticides, but there was some overlap with our study because they used organophosphates (n = 10, 8.9% of all pesticides used).^30^

Researchers in SE Iran completed a case control study of 141 family members of farmworkers focused on organophosphate and organic chlorine pesticides.^31^ It was found that AChE activity was significantly decreased compared with control subject (*P* < .001).^31^ This study also found an inverse relationship with paraoxonase 1, superoxide dismutase, glutathione peroxidase, and total antioxidant capacity amount, which suggest epigenetic change and oxidative stress among farmworkers.^31^

In a study by Sapbamrer and Nata, rice farmers and non‐farmer controls residing in Northern Thailand were interviewed and had blood samples taken to ascertain their overall pesticide exposure.^32^ When looking at self‐reported health outcomes in our study, farmworkers did not have differences in respiratory symptoms relative to comparison workers. Farmworkers reported trembling in their hands less often in comparison with other workers; however, with exposure to AChE inhibitors, we would expect farmworkers would report trembling more often.

### 4.3 Health biomarkers and symptoms

When trying to assess cholinesterase changes due to chemical exposure, it is standard to take pre‐ and post‐spraying samples to ascertain measurement alterations before and after exposure to pesticides.^18^, ^33^, ^34^, ^35^, ^36^, ^37^ The Washington Farmers Study uses a definition of 20% cholinesterase depression to be clinically significant cholinesterase depression and is often used as the golden standard for many study looking at cholinesterase depression in farmworkers.^37^ The Washington Cholinesterase Monitoring Program takes one baseline sample prior to spraying and one follow‐up sample taken at one or more follow‐up visits after a suspected exposure to organophosphates.^18^, ^37^ In Quandt et al, researchers assessed the health of 231 migrant farmworkers (H‐2A US Visa) residing across 11 counties in North Carolina and took four blood collections to determine cholinesterase depression.^38^ In the Australian Cholinesterase Research Outreach Project (CROP), researchers analyzed cholinesterase depression among farmworkers and non‐farmworkers residing in South West Victoria and took one baseline sample and three subsequent samples at different times thought to be high post‐exposure times.^34^

Among cotton farmers in Pakistan, 82% of farmers reported a health impairment with the most common symptoms being irritation of skin and eyes, headache, and dizziness.^39^ These symptoms did not appear within 24 hours prior to spraying pesticides, and the average amount of multiple symptoms is 2.6 ± 0.88.^39^ Studies that also looked at DNA damage and liver function biomarkers found that farmers in Pakistan were exposed to five different chemicals including chlorpyrifos‐methyl, carbosulfan, profenofos, cypermethrin, endosulfan sulfate.^35^ In a study that looks at serum cholinesterase as a function of liver function, found slightly lower serum cholinesterase levels compared with non‐industry/pesticide workers (69.2% vs. 19.2%, respectively).^35^ García‐García et al completed a similar study comparing 169 green house workers who were exposed to pesticides and controls who were unexposed to pesticides in southeastern Spain.^40^ Among this cohort, researchers controlled for sex, age, and BMI, but not smoking status. They found BuChE inhibition, RBC, MCV, and neutrophil levels significantly increased, whereas eosinophils significantly decreased and monocytes were not different.^40^

Our study assessed hematological parameters by measuring differences in CBCs between farmworkers and comparison workers, while controlling for BMI, age, former smoker status, current smoker status, worker category, and pesticide exposure levels. Serum calcium was statistically significant by both worker category and was also significantly different in farmworker. MCV, monocytes, RBC, eosinophils, urine creatinine and WBC were significantly different between our Northern Thailand farmworkers and comparison workers. In our study, farmworkers had a reduction, in RBC and neutrophils, whereas MCV increased (opposite of García‐García et al). Monocytes and eosinophils reduced and increased, respectively, in our study and the García‐García et al study.^40^ In our study, an association existed between pesticide exposure and all the aforementioned blood counts when looking at farmworkers and comparison workers.

Finally, a cohort study on farmworkers in China before and after pesticide spraying points to the effects of pesticides on CBC having an effect that can be categorized as long term (3 years) or short term (10 days or less).^41^ Long‐term results include decreased white blood cell count.^41^ Short‐term results include as alterations in CBC; hepatic and renal functions; nerve conduction velocities; and on monocytes, hemoglobin, and platelet counts.^41^ When comparing air sample measurements of methomyl, chlorpyrifos and metalaxyl, we did not identify differences in blood count analytes by chemical exposure, although we identified a range of differences when comparing farmworkers and comparison workers. Prior research has reported significant changes to hematological biochemistry as a result of pesticide exposures causing oxidative stress.^40^ Oxidative stress due to pesticide spraying has also been related to changes in CBC such as monocytosis, leukocytosis, neutrophilia, and lymphocytopenia, which were thought to also be closely related to patients in oxidative stress.^40^, ^42^

### 4.4 Strengths and limitations

Our study has some limitations. We focused on farmworkers who contracted to numerous farms, and therefore, workers were likely exposed to other chemicals that were not quantified in this study. These farmworkers may have been exposed to chemicals that were not captured by this study because they sprayed pesticides on other farms and sprayed other plants on the same farms. Due to this discrepancy, our farmworkers' baseline cholinesterase measurements may not actually represent a true baseline measurement due to their overlapping work schedules. The US Occupational Safety and Health Administration (OSHA) generally recommends testing for baseline cholinesterase levels after not working with organophosphates for at least 30 days.^43^ OSHA also recommends taking a baseline measurement before and after organophosphate use (with at least a 30 day washout period for both).^43^ This pilot study did not take multiple baseline measurements, and the one baseline that was taken was likely taken before the OSHA recommended guideline of 30 days because pesticide use. However, due to the crop production schedule and growing seasons in Northern Thailand, this population does not appear to ever experience thirty days of no pesticide use. Our study also focused on workplace sampling at a time when the specific farm of interest was expected to be spraying chlorpyrifos; therefore, the study results show an over‐representation of chlorpyrifos.

Overall, this is the first study of its type that took a mixed‐methods approach using survey, biomarker, and workplace observation data to analyze farmworker pesticide health effects in comparison to other workers in Northern Thailand. Additionally, this pilot study is one of the larger studies on farmworker chemical exposures in Thailand. These data can inform the methods for future global occupational health research on farmworkers. Work observations also included a more detailed outline of PPE used by the farmworkers and could inform future studies on PPE in relation to pesticide exposure and preventive interventions. This study is very generalizable to farmworkers in LMIC and Thailand. This study will contribute to the building literature on farmworker pesticide exposure and resultant health outcomes.

## DISCLOSURES

*Ethical approval*: The study protocol was approved by the Institutional Review Boards of Mae Fah Luang University and the University of Michigan. *Informed consent*: All participants provided oral consent to participate in the study which was noted by the researcher administering the survey in the first step of study participation. *Registry and the registration no. of the study/trial*: The study protocol was approved by the Institutional Review Boards of Mae Fah Luang University and the University of Michigan (UM Submission ID: HUM00128091/CR00068767). *Animal studies*: N/A. *Conflict of interest*: N/A.

## Supporting information

Table S1

Table S2

Table S3
