# Supplementary material for: Pesticide exposure and adverse health effects associated with farmwork in Northern Thailand
Source: J Occup Health. 2021 May 11;63(1):e12222. doi: 10.1002/1348-9585.12222 (PMC8112117; doi:10.1002/1348-9585.12222)
Supplement: Supplementary file 1 — Table S1 [file JOH2-63-e12222-s002.docx]

| Supplemental Table 1 | | | | | | | | | | | | | |
| --- | --- | --- | --- | --- | --- | --- | --- | --- | --- | --- | --- | --- | --- |
| Unadjusted Linear regression models of association between blood analytes and pesticide air sample concentrations | | | | | | | | | | | | | |
|  |  |  |  |  |  |  |  |  |  |  |  |  |  |
| Analytes | Units | Log(Methomyl) | | | | Log(Metalaxyl) | | | | Log(Ethyl Chlorpyrifos) | | | |
|  |  | β | p-value | 95% CI | | β | p-value | 95% CI | | β | p-value | 95% CI | |
| log(Basophil) | % | 0.18 | 0.68 | -0.70 | 1.06 | -0.05 | 0.52 | -0.22 | 0.12 | 0.03 | 0.67 | -0.13 | 0.20 |
| log(Eosinophil) | % | 0.18 | 0.72 | -1.14 | 0.79 | 0.00 | 0.96 | -0.19 | 0.18 | -0.02 | 0.82 | -0.20 | 0.16 |
| log(Hemoglobin) | g/dl | -0.01 | 0.70 | -0.09 | 0.06 | 0.01 | 0.38 | -0.01 | 0.02 | 0.01 | 0.18 | 0.00 | 0.02 |
| log(Hematocrit) | % | -0.04 | 0.30 | -0.12 | 0.04 | 0.01 | 0.43 | -0.01 | 0.02 | 0.01 | 0.47 | -0.01 | 0.02 |
| log(Lymphocyte) | % | 0.12 | 0.48 | -0.22 | 0.46 | -0.01 | 0.68 | -0.08 | 0.05 | -0.05 | 0.12 | -0.11 | 0.01 |
| log(MCHC) | pg | 0.03 | 0.25 | -0.02 | 0.08 | 0.00 | 0.97 | -0.01 | 0.01 | 0.00 | 0.47 | -0.01 | 0.01 |
| log(MCV) | fl | -0.06 | 0.40 | -0.20 | 0.08 | 0.02 | 0.16 | -0.01 | 0.05 | 0.02 | 0.24 | -0.01 | 0.04 |
| Monocyte | % | -1.54 | 0.48 | -5.91 | 2.83 | 0.06 | 0.88 | -0.79 | 0.92 | -0.18 | 0.66 | -1.00 | 0.64 |
| Neutrophil | % | 0.98 | 0.95 | -31.15 | 33.12 | 1.51 | 0.62 | -4.71 | 7.73 | 3.90 | 0.18 | -1.88 | 9.68 |
| log(Plate Count) | cells/uL | 0.05 | 0.72 | -0.22 | 0.32 | -0.03 | 0.29 | -0.08 | 0.02 | -0.05 | 0.02 | -0.10 | -0.01 |
| log(RBC) | cells/uL | 0.02 | 0.82 | -0.14 | 0.17 | -0.01 | 0.39 | -0.04 | 0.02 | -0.01 | 0.49 | -0.04 | 0.02 |
| log(RDW) | % | 0.05 | 0.26 | -0.04 | 0.13 | 0.00 | 0.74 | -0.02 | 0.01 | 0.00 | 0.62 | -0.02 | 0.01 |
| log(WBC) | cells/uL | -0.03 | 0.82 | -0.27 | 0.22 | 0.00 | 0.93 | -0.05 | 0.05 | -0.01 | 0.80 | -0.05 | 0.04 |
| Serum Calcium | mg/dl | 0.08 | 0.73 | -0.39 | 0.55 | -0.40 | 0.07 | -0.83 | 0.03 | 0.15 | 0.53 | -0.33 | 0.62 |
| log(Serum Creatinine) | mg/dl | -0.02 | 0.18 | -0.06 | 0.01 | -0.01 | 0.44 | -0.05 | 0.02 | 0.02 | 0.33 | -0.02 | 0.05 |
| log(Urine Calcium) | mg/dl | 0.02 | 0.78 | -0.12 | 0.16 | -0.02 | 0.73 | -0.15 | 0.11 | 0.09 | 0.21 | -0.05 | 0.22 |
| Urine Creatinine | mg/dl | 5.17 | 0.67 | -19.35 | 29.69 | -18.99 | 0.09 | -41.37 | 3.39 | 9.23 | 0.45 | -15.36 | 33.83 |
| AChE Ratio^b^ | U/L | -0.31 | 0.05 | -0.62 | -0.01 | 0.06 | 0.71 | -0.24 | 0.35 | 0.16 | 0.31 | -0.16 | 0.48 |
| BuChE Ratio^b^ | U/L | -0.06 | 0.62 | -0.33 | 0.20 | -0.06 | 0.63 | -0.30 | 0.19 | -0.15 | 0.26 | -0.41 | 0.11 |
| ^a^Signifies a p-value less than 0.05 | |  |  |  |  |  |  |  |  |  |  |  |  |
| ^b^Cholinesterase ratios were calculated by dividing post measures by pre measures. | | | | | |  |  |  |  |  |  |  |  |
