# Supplementary material for: Pesticide exposure and adverse health effects associated with farmwork in Northern Thailand
Source: J Occup Health. 2021 May 11;63(1):e12222. doi: 10.1002/1348-9585.12222 (PMC8112117; doi:10.1002/1348-9585.12222)
Supplement: Supplementary file 2 — Table S2 [file JOH2-63-e12222-s003.docx]

| Supplemental Table 2  Northern Thailand Participant Self-reported Symptoms by worker category | | | | | | |
| --- | --- | --- | --- | --- | --- | --- |
|  | Variable Outcome | Comparison Worker | | Farmworker | | Fisher's Exact Test |
|  |  | N=27 | % | N=70 | % | p-value |
| In the last two weeks, how often have you had the following conditions… | | | | | |  |
| stuffy, itchy, runny nose? | rarely or never | 18 | 66.7 | 51 | 72.9 | 0.31 |
|  | occasionally | 8 | 29.6 | 15 | 21.4 |  |
|  | frequently | -- | -- | 2 | 2.9 |  |
| watery, itchy eyes? | rarely or never | 20 | 74.1 | 47 | 67.1 | 0.12 |
|  | occasionally | 4 | 14.8 | 20 | 28.6 |  |
|  | frequently | 2 | 7.4 | 2 | 2.9 |  |
| sinusitis or sinus problems? | rarely or never | 25 | 92.6 | 68 | 97.1 | 0.19 |
|  | occasionally | 1 | 3.7 | --- | -- |  |
|  | frequently | -- | -- | 1 | 1.4 |  |
| pneumonia? | rarely or never | 26 | 96.3 | 62 | 88.6 | 0.14 |
|  | occasionally | -- | -- | 5 | 7.1 |  |
|  | frequently | -- | -- | 3 | 4.3 |  |
| dizziness? | rarely or never | 23 | 85.2 | 45 | 64.3 | 0.03 |
|  | occasionally | 3 | 11.1 | 23 | 32.9 |  |
|  | frequently | -- | -- | 1 | 1.4 |  |
| nausea/vomiting? | rarely or never | 25 | 92.6 | 61 | 87.1 | 0.2 |
|  | occasionally | 1 | 3.7 | 8 | 11.4 |  |
|  | frequently | -- | -- | -- | -- |  |
| Being absentminded, forgetful, or confused? | rarely or never | 12 | 44.4 | 31 | 44.3 | 0.55 |
|  | occasionally | 11 | 40.7 | 29 | 41.4 |  |
|  | frequently | 3 | 11.1 | 10 | 14.3 |  |
| headache? | rarely or never | 17 | 63.0 | 48 | 68.6 | 0.4 |
|  | occasionally | 9 | 33.3 | 20 | 28.6 |  |
|  | frequently | -- | -- | 1 | 1.4 |  |
| loss of appetite? | rarely or never | 23 | 85.2 | 62 | 88.6 | 0.38 |
|  | occasionally | 3 | 11.1 | 7 | 10.0 |  |
|  | frequently | -- | -- | -- | -- |  |
| fast heart rate? | rarely or never | 25 | 92.6 | 57 | 81.4 | 0.14 |
|  | occasionally | 1 | 3.7 | 10 | 14.3 |  |
|  | frequently | -- | -- | 1 | 1.4 |  |
| difficulty with balance? | rarely or never | 21 | 77.8 | 59 | 84.3 | 0.24 |
|  | occasionally | 5 | 18.5 | 10 | 14.3 |  |
|  | frequently | -- | -- | -- | -- |  |
| blurred vision or double vision? | rarely or never | 16 | 59.3 | 41 | 58.6 | 0.15 |
|  | occasionally | 9 | 33.3 | 25 | 35.7 |  |
|  | frequently | -- | -- | 3 | 4.3 |  |
| numbness or pins-and-needles in your hands or feet? | rarely or never | 15 | 55.6 | 41 | 58.6 | 0.32 |
|  | occasionally | 11 | 40.7 | 25 | 35.7 |  |
|  | frequently | -- | -- | 4 | 5.7 |  |
| shaking or trembling of your hands? | rarely or never | 18 | 66.7 | 61 | 87.1 | 0.01 |
|  | occasionally | 8 | 29.6 | 6 | 8.6 |  |
|  | frequently | -- | -- | 1 | 1.4 |  |
| twitches, jerks, or involuntary movements of your arms or legs? | rarely or never | 20 | 74.1 | 54 | 77.1 | 0.39 |
|  | occasionally | 6 | 22.2 | 12 | 17.1 |  |
|  | frequently | -- | -- | 1 | 1.4 |  |
