# Supplementary material for: Pesticide exposure and adverse health effects associated with farmwork in Northern Thailand
Source: J Occup Health. 2021 May 11;63(1):e12222. doi: 10.1002/1348-9585.12222 (PMC8112117; doi:10.1002/1348-9585.12222)
Supplement: Supplementary file 3 — Table S3 [file JOH2-63-e12222-s001.docx]

| Supplemental Table 3  Northern Thailand Participant Stressor Responses 2017, by worker category | | | | | | |
| --- | --- | --- | --- | --- | --- | --- |
| Variable | Variable Outcome | Comparison Worker | | Farmworker | | Fisher's Exact Test |
|  |  | N=27 | % | N=70 | % | p-value |
| In the last month, how often have you felt that you were unable to control the important things in your life? | never | 17.0 | 63.0 | 48.0 | 68.6 | 0.57 |
|  | almost never | 4.0 | 14.8 | 13.0 | 18.6 |  |
|  | sometimes | 5.0 | 18.5 | 8.0 | 11.4 |  |
|  | fairly often | 1.0 | 3.7 | 1.0 | 1.4 |  |
|  | Often | -- | -- | -- | -- |  |
| In the last month, how often have you felt confident about your ability to handle your personal problems? | never | -- | -- | 4.0 | 5.7 | 0.03 |
|  | almost never | -- | -- | 2.0 | 2.9 |  |
|  | sometimes | 2.0 | 7.4 | 6.0 | 8.6 |  |
|  | fairly often | 4.0 | 14.8 | 28.0 | 40.0 |  |
|  | Often | 21.0 | 77.8 | 30.0 | 42.9 |  |
| In the last month, how often have you felt that things were going your way? | never | -- | -- | -- | -- | 0.27 |
|  | almost never | -- | -- | 1.0 | 1.4 |  |
|  | sometimes | 8.0 | 29.6 | 28.0 | 40.0 |  |
|  | fairly often | 6.0 | 22.2 | 22.0 | 31.4 |  |
|  | Often | 13.0 | 48.2 | 19.0 | 27.1 |  |
| In the last month, how often have you felt difficulties were piling up so high that you could not overcome them? | never | 16.0 | 59.3 | 49.0 | 70.0 | 0.34 |
|  | almost never | 8.0 | 29.6 | 11.0 | 15.7 |  |
|  | sometimes | 3.0 | 11.1 | 10.0 | 14.3 |  |
|  | fairly often | -- | -- | -- | -- |  |
|  | Often | -- | -- | -- | -- |  |
|  | range | median |  | standard deviation | | |
| How many hours do you work in a typical week in total at all of your jobs? | 6-140 | 50.96 | | 25.73 | | |
